# Interplay between FACT subunit SPT16 and TRIM33 can remodel chromatin at macrophage distal regulatory elements

**DOI:** 10.1186/s13072-019-0288-3

**Published:** 2019-07-22

**Authors:** Federica Ferri, Vanessa Petit, Vilma Barroca, Paul-Henri Romeo

**Affiliations:** 1 CEA/DRF/IBFJ/iRCM/LRTS, 92265 Fontenay-aux-Roses Cedex, France; 2 Inserm U967, 92265 Fontenay-aux-Roses Cedex, France; 3 Université Paris-Diderot, Paris 7, France; 4 Université Paris-Sud, Paris 11, France; 5 Equipe labellisée Ligue contre le Cancer, Paris, France

**Keywords:** Macrophage, Distal regulatory elements, Nucleosome positioning, FACT, TRIM33

## Abstract

**Background:**

Cell type-specific use of *cis*-acting regulatory elements is mediated by the combinatorial activity of transcription factors involved in lineage determination and maintenance of cell identity. In macrophages, specific transcriptional programs are dictated by the transcription factor PU.1 that primes distal regulatory elements for macrophage identities and makes chromatin competent for activity of stimuli-dependent transcription factors. Although the advances in genome-wide approaches have elucidated the functions of these macrophage-specific distal regulatory elements in transcriptional responses, chromatin structures associated with PU.1 priming and the underlying mechanisms of action of these *cis*-acting sequences are not characterized.

**Results:**

Here, we show that, in macrophages, FACT subunit SPT16 can bind to positioned nucleosomes directly flanking PU.1-bound sites at previously uncharacterized distal regulatory elements located near genes essential for macrophage development and functions. SPT16 can interact with the transcriptional co-regulator TRIM33 and binds to half of these sites in a TRIM33-dependent manner. Using the *Atp1b3* locus as a model, we show that FACT binds to two positioned nucleosomes surrounding a TRIM33/PU.1-bound site in a region, located 35 kb upstream the *Atp1b3* TSS, that interact with the *Atp1b3* promoter. At this − 35 kb region, TRIM33 deficiency leads to FACT release, loss of the two positioned nucleosomes, RNA Pol II recruitment and bidirectional transcription. These modifications are associated with higher levels of FACT binding at the *Atp1b3* promoter, an increase of RNA Pol II recruitment and an increased expression of *Atp1b3* in *Trim33*^−/−^ macrophages.

**Conclusions:**

Thus, sequestering of SPT16/FACT by TRIM33 at PU.1-bound distal regions might represent a new regulatory mechanism for RNA Pol II recruitment and transcription output in macrophages.

**Electronic supplementary material:**

The online version of this article (10.1186/s13072-019-0288-3) contains supplementary material, which is available to authorized users.

## Background

Cell type-specific transcriptional programs involve the selection and activation of distal regulatory elements by the combinatorial activity of lineage-determining transcription factors and chromatin modifiers, nucleosome remodelers and histone chaperons that modify functionality and accessibility of the underlying DNA sequences. Genome-wide approaches have indicated the mechanisms underlying the selection and the control of the timing and the magnitude of enhancer activation in transcriptional responses [1]. Nucleosomes directly flanking transcription factor binding sites at enhancers bear specific histone modifications, including, but not limited to, enrichment of H3K4me1 or H3K4me2. In their active state, enhancers are additionally marked by H3K27ac and are bound by general transcription factors and RNA Pol II, leading to the production of enhancer-originating RNA (eRNA) [2]. In contrast to this advanced knowledge, little is known about the mechanisms that regulate the activity of *cis*-acting regulatory regions that are not enhancer.

Macrophages exhibit functional diversity and ability to undergo rapid reprogramming depending on their microenvironment, and this plasticity is driven by functionally diversified *cis*-regulatory elements. The transcription factor PU.1 binds most of these *cis*-acting regulatory elements and, in combination with other transcriptional regulators such as C/EBPα or RUNX1, promotes the creation of a macrophage-specific enhancer landscape. In addition, binding of PU.1 makes chromatin competent for binding and activity of stimuli-dependent transcription factors, such as NF-κB, AP-1, STAT or IRF family members [3, 4].

We previously identified the transcriptional regulator TRIM33 as an important co-repressor of PU.1 activity in myeloid cells [5]. TRIM33 belongs to a subfamily of TRIM proteins characterized by a chromatin-binding PHD/BROMO domain [6]. TRIM33 cannot directly bind to DNA but, depending on the cellular and signaling context, can be recruited by cell type-specific transcription factors to act as a transcriptional co-activator [7, 8] or co-repressor [5, 9–12]. Through its binding to PU.1-bound regulatory regions, TRIM33 regulates the production of macrophages and is involved in the innate immune response by repressing a subset of genes regulating the late stages of lipopolysaccharide activation of macrophages [10]. At the molecular level, TRIM33 switches off *Ifnb1* gene transcription during the late phase of macrophage activation by preventing recruitment of the co-activator p300 [9].

The histone chaperone FAcilitates Chromatin Transcription (FACT) is a heterodimer of SPT16 and SSRP1 proteins, highly conserved among eukaryotes. FACT was initially identified as essential for transcriptional elongation through chromatin templates [13]. Associated with the RNA Pol II elongating complex, FACT is important in reversing the nucleosome disruption that occurs upon elongation, thereby suppressing inappropriate initiation from cryptic promoters within coding regions [14, 15]. Through a dual function in breaking the nucleosome and maintaining its integrity [16], FACT has been shown to play a pivotal role in almost all chromatin-related processes, including transcription, replication, recombination and repair [17–23]. While the general role of FACT in reorganizing nucleosomes is well characterized, it is not known so far whether FACT is involved in the dynamic remodeling of chromatin at gene distal regulatory elements.

Here, we show that the FACT subunit SPT16 binds on positioned nucleosomes flanking PU.1-occupied sites in proximity of genes essential for macrophage development and function. We provide evidence for the interplay between FACT and TRIM33 in maintaining a stable chromatin structure at a PU.1-bound distal regulatory region to prevent non-coding transcription and to limit nearby gene transcription.

## Results

### FACT subunit SPT16 is bound to distal regulatory regions of genes essential for macrophage development and function

**Fig. 1:**
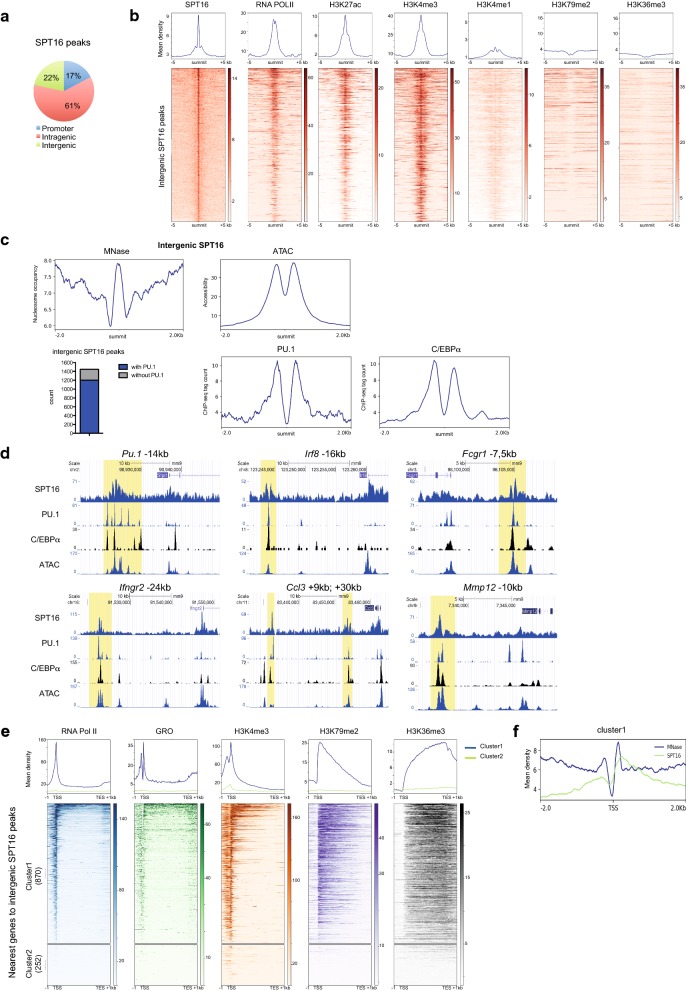
SPT16 binding at macrophage distal regulatory regions. **a** Genomic distribution of SPT16 peaks in BMDM. **b** SPT16, RNA Pol II and indicated histone modifications profiles at the summit of intergenic SPT16 peaks ± 5 kb. H3K27ac and H3K4me1 ChIP-seq were retrieved from ENCODE. Regions were ranked based on decreasing SPT16 signals. **c** (Up) Average MNase and ATAC profiles at the summit of intergenic SPT16 peaks ± 2 kb. (Bottom) Overlap between PU.1 and intergenic SPT16 peaks (left). Average PU.1 (middle) and C/EBPα (right) profiles at the summit of intergenic SPT16 peaks ± 2 kb. **d** Representative examples for SPT16 occupancy along with PU.1, C/EBPα and ATAC-seq profiles at distal regulatory regions. Regions corresponding to intergenic SPT16 peaks are highlighted. **e** RNA Pol II, GRO-seq and indicated chromatin marks profiles at bodies of genes associated with intergenic SPT16 peaks. Genes were clustered into two major categories based on decreasing RNA Pol II levels. **f** Nucleosome occupancy and SPT16 profile at the TSS of cluster1 genes

Genome-wide occupancy of FACT subunit SPT16 in mouse bone marrow-derived macrophages (BMDM) was investigated by ChIP-sequencing (ChIP-seq) that identified 7943 SPT16-occupied regions. 78% of the SPT16 peaks were located in intragenic regions and promoters (Fig. 1a). In accordance with the role of FACT in transcription elongation [13, 17], most of these SPT16 peaks co-localized with RNA Pol II occupancy (Additional file 1: Figure S1a). The other SPT16 peaks, located in intergenic regions, were sharp (Additional file 1: Figure S1a), associated with enrichment of RNA Pol II, H3K27ac, H3K4me3 and, to a lesser extent, H3K4me1 histone marks but not with H3K79me2 and H3K36me3 histone marks (Fig. 1b) suggesting that these intergenic SPT16 peaks defined regulatory regions. Integrating data of nucleosome positioning obtained with Micrococcal Nuclease digestion (MNase-seq) [24] and Assay for Transposase Accessible Chromatin (ATAC-seq) in BMDM, we show that, in a 4 kb region centered on the intergenic SPT16 summit peaks, SPT16 binding occurred at positioned nucleosomes flanked by nucleosome-depleted regions that can bind *trans*-acting factors (Fig. 1c, upper panels). In accordance, PU.1, which bound to most of macrophage-specific regulatory regions, co-localized with SPT16 in 83% of these intergenic regions (Fig. 1c, lower left panel) and PU.1 was bound at the nucleosome-depleted regions flanking the SPT16 peak (Fig. 1c, lower middle panel). A similar enrichment was observed for the myeloid-restricted transcription factor C/EBPα (Fig. 1c, lower right panel), an important PU.1 interacting factor in macrophages [4].

Genes located near these peaks were involved in transcription regulation and innate immune response (Additional file 1: Figure S1b). They included key macrophage transcription factors such as *Irf8*, *Cebpα* and *Esr1*, well-characterized macrophage surface markers such as *Fcgr1* and Toll-like receptors, receptors for interferon gamma and alpha (*Ifngr2* and *Ifnar2*), chemokines such as *Ccl3* and *Mmp12*, a member of the matrix metalloproteinase family (Fig. 1d) [25]. Moreover, SPT16 was bound to nucleosomes surrounding several PU.1-occupied sites in a region known to confer myeloid-specific PU.1 expression (Fig. 1d) [26]. SSRP1, the other subunit of FACT, was also bound to SPT16-marked intergenic regions suggesting that the FACT complex was bound to these regions (Additional file 1: Figure S1c). Integrating RNA Pol II ChIP-seq enrichment profiles showed that most of these SPT16 tagged genes (> 75%) had a high level of RNA Pol II at gene bodies (Fig. 1e), and GRO-seq analysis from published data [27] associated these RNA Pol II enrichments and active transcription (Fig. 1e). SPT16 tagged genes also displayed high enrichment of histone mark H3K4me3, highly enriched at transcription start site (TSS) of active promoters, and H3K79me2 and H3K36me3 marks, two histone marks enriched at actively transcribed gene bodies and associated with RNA Pol II phosphorylation [28] (Fig. 1e). Finally, metaprofiles of SPT16 binding at TSS of these genes showed preferential association with the first (+ 1) downstream nucleosome (Fig. 1f and Additional file 1: Figure S1d). Altogether, these results show that SPT16 is bound on positioned nucleosomes surrounding PU.1-bound sites at specific macrophage regulatory regions that were previously uncharacterized.

### SPT16 recruitment at macrophage distal regulatory regions can be regulated by TRIM33

**Fig. 2:**
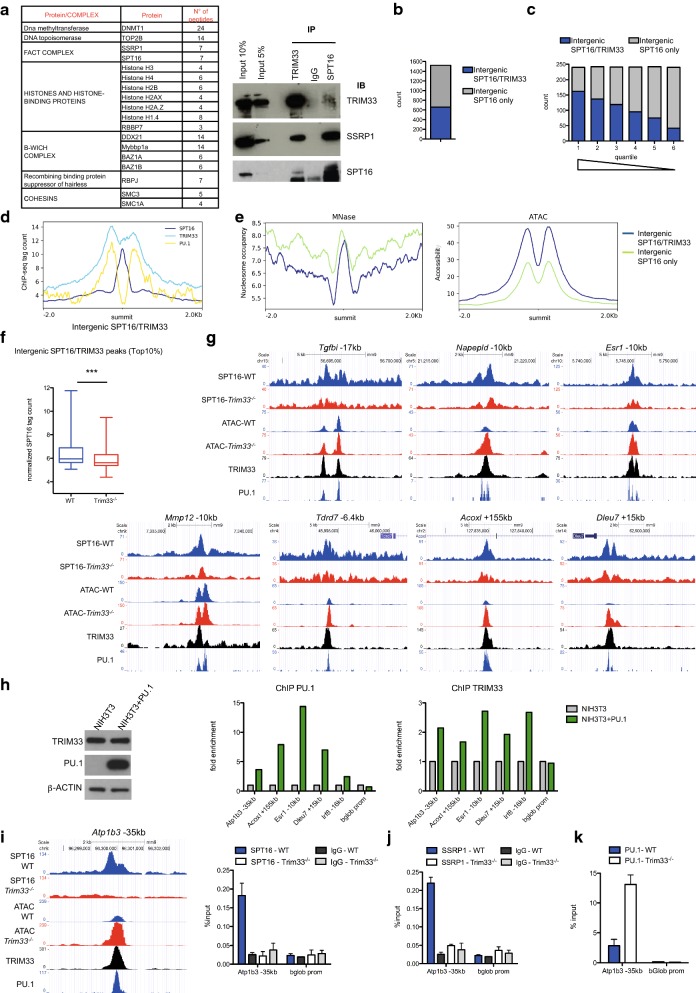
TRIM33-dependent SPT16 recruitment at macrophage distal regulatory regions. **a** (Left) Proteins and number of peptides identified following Flag-TRIM33 immunopurification and mass spectrometry in immortalized macrophages (IM). (Right) Immunoprecipitation (IP) of endogenous proteins from IM nuclear extracts followed by immunoblotting (IB). **b** Overlap between intergenic SPT16 and TRIM33 peaks in BMDM. **c** Intergenic SPT16 peaks were divided in quantiles based on their tag density. Higher enriched intergenic SPT16 peaks have higher number of peaks that co-localize with TRIM33 peaks. **d** TRIM33, PU.1 and SPT16 occupancy at the summit of intergenic SPT16/TRIM33 peaks ± 2 kb. **e** MNase (left) and ATAC (right) profiles at the summit of intergenic SPT16 peaks that co-localized (SPT16/TRIM33) or not (SPT16 only) with TRIM33. **f** Normalized SPT16 ChIP-seq tag count at most enriched (Top10%) intergenic SPT16/TRIM33 peaks in WT and *Trim33*^−/−^ BMDM. ***p < 0.0001, Paired *t* test. **g** SPT16 ChIP-seq and ATAC-seq profiles at indicated loci in WT and *Trim33*^−/−^ BMDM along with TRIM33 and PU.1 occupancy in WT BMDM. **h** Immunoblotting of TRIM33, PU.1 and β-ACTIN in NIH3T3 and NIH3T3 transfected with PU.1 (left). PU.1 (middle) and TRIM33 (right) ChIP-qPCR at indicated loci in NIH3T3 and NIH3T3 transfected with PU.1. **i** SPT16 ChIP-seq (left) and ChIP-qPCR (right) at the *Atp1b3* −35 kb region in WT and *Trim33*^−/−^ BMDM. Mean ± SEM, *n* = 3. **j** SSRP1 and **k** PU.1 ChIP-qPCR at the *Atp1b3* − 35 kb region in WT and *Trim33*^−/−^ BMDM. Mean ± SEM, *n* = 3

FACT interacts with the transcriptional co-regulator TRIM33 in K562 human erythroleukemia [7] and Hela cells [29]. As TRIM33 binds to several PU.1-occupied sites in BMDM and is an important regulator of macrophage production and activation [9, 10], we asked whether TRIM33 could form a complex with FACT in BMDM and if this complex is bound to the intergenic SPT16-occupied regulatory regions. Flag-tagged TRIM33 was stably expressed at a level similar to that of endogenous TRIM33 in *Trim33*^−/−^ immortalized macrophages [9]. Nuclear extracts were immunoprecipitated using a FLAG antibody and analyses of the precipitated proteins identified the two subunits of the FACT complex, SPT16 and SSRP1, along with numerous histones and histone-binding proteins (Fig. 2a, left table). The interaction between endogenous TRIM33 and the FACT complex was shown by co-immunoprecipitation (Fig. 2a, right panel).

SPT16 and TRIM33 ChIP-seq analyses in BMDM showed that half of SPT16 peaks co-localized with TRIM33 peaks (Additional file 2: Figure S2a). 43% of the SPT16 intergenic peaks co-localized with TRIM33 peaks (Fig. 2b). These peaks displayed high SPT16 read densities (Fig. 2c) and were located near genes involved in immune system processes (Additional file 2: Figure S2b). At SPT16/TRIM33 intergenic regions, TRIM33 was located around the summit of SPT16 peaks in a pattern similar to PU.1 binding (Fig. 2d) and SPT16 binding occurred to more positioned nucleosomes (Fig. 2e). SPT16 ChIP-seq in *Trim33*^−/−^ BMDM showed that, although deletion of *Trim33* did not modify global SPT16 binding (Additional file 2: Figure S2c; Spearman coefficient 0.91), SPT16 tag coverage decreased in *Trim33*^−/−^ BMDM at intergenic SPT16/TRIM33 peaks but not at intergenic SPT16 peaks not co-occupied by TRIM33 (Fig. 2f, g and Additional file 2: Figure S2d). A decrease in SSRP1 binding was also found at several regions where SPT16 binding is decreased in *Trim33*^−/−^ BMDM (Additional file 2: Figure S2e) suggesting an impaired FACT binding at these regions in *Trim33*^−/−^ BMDM. This decrease was not associated with decreased PU.1 binding at these sites (Additional file 2: Figure S2f for examples) and was rescued by exogenous expression of TRIM33 in *Trim33*^−/−^ immortalized macrophages (Additional file 2: Figure S2g for examples), indicating that TRIM33 but not PU.1 might regulate SPT16 recruitment at these intergenic regions in BMDM. We addressed the role of PU.1 on the recruitment of TRIM33 at intergenic SPT16/TRIM33 co-occupied regions. As depletion of PU.1 is not compatible with primary macrophage survival [24], we first transfected Hela cells with expression vectors encoding Flag-TRIM33 and PU.1 along with a reporter plasmid containing three PU.1 binding sites [5]. ChIP analyses showed a PU.1-dependent recruitment of Flag-TRIM33 at these PU.1 sites (Additional file 2: Figure S2h). To address TRIM33 recruitment by PU.1 in chromatin, we transiently expressed PU.1 in NIH3T3 cells, that express TRIM33 but not PU.1 (Fig. 2h, left panel), and studied PU.1 and TRIM33 binding to several known PU.1/TRIM33 bound loci in BMDM. Exogenous expression of PU.1 in NIH3T3 cells resulted in PU.1 binding to these loci (Fig. 2h, middle panel) and TRIM33 binding to these loci occurred only in presence of PU.1 (Fig. 2h, right panel), strengthening a PU.1-dependent recruitment of TRIM33 to chromatin in BMDM.

ATAC-Seq was used to study the effect of TRIM33 deficiency on chromatin accessibility in BMDM. TRIM33 deficiency did not alter genome-wide chromatin accessibility (284 regions more opened and 209 more closed in *Trim33*^−/−^ compared to WT BMDM, log2FoldChange ≥ 1, adjusted *p* value ≤ 0.05, out of 118,434 regions identified). However, analyses of distal SPT16/TRIM33 regions, in which SPT16 was abrogated in *Trim33*^−/−^ BMDM, showed a higher accessibility in *Trim33*^−/−^ compared to WT BMDM (Fig. 2g), suggesting a role of SPT16/TRIM33 in chromatin organization at these loci.

The most striking effect of TRIM33 deficiency on SPT16 occupancy and chromatin accessibility was observed at a TRIM33/PU.1-bound region located 35 kb upstream the TSS of the *Atp1b3* gene (or 79 kb downstream the TSS of the *Rnf7* gene) (Additional file 2: Figure S2i). At this locus, SPT16 was bound in WT BMDM with two SPT16 peaks surrounding the TRIM33/PU.1 peak (Fig. 2i, left panel). TRIM33 deficiency abolished SPT16 binding (Fig. 2i, left and right panels), SSRP1 binding (Fig. 2j) but not PU.1 binding that was even increased (Fig. 2k), along with a gain in chromatin accessibility (Fig. 2i). These results pinpointed the *Atp1b3* locus as a model to study the interplay between FACT and TRIM33 at distal regulatory regions in macrophages.

### FACT/TRIM33 interaction at the − 35 kb regulatory region modulates RNA Pol II recruitment at the *Atp1b3* promoter

**Fig. 3:**
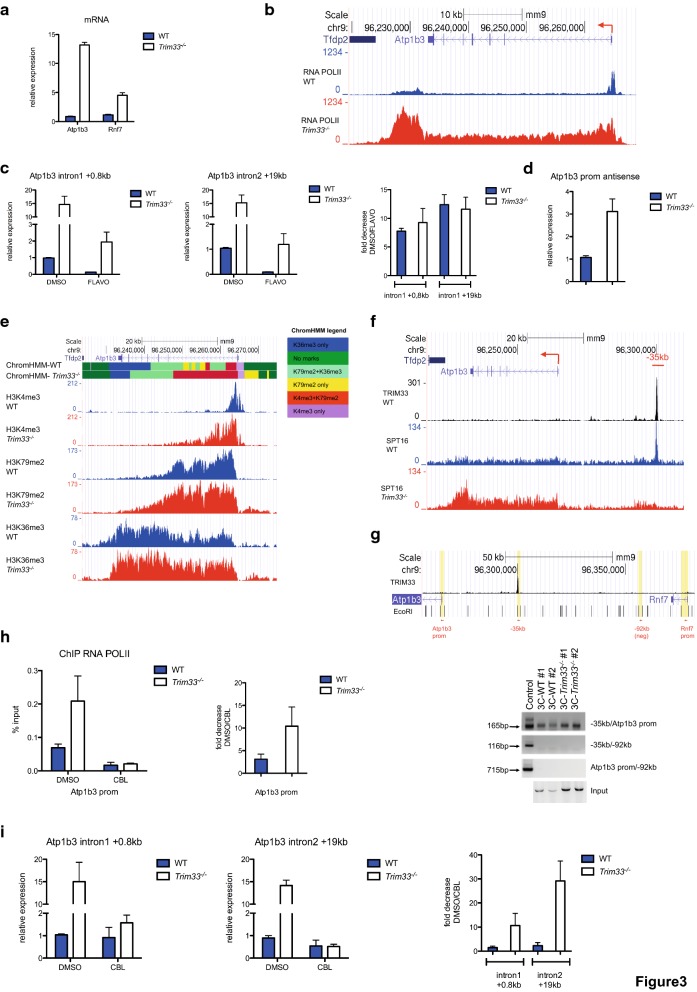
RNA Pol II recruitment at the *Atp1b3* promoter is regulated by FACT/TRIM33. **a** *Atp1b3* and *Rnf7* mRNA levels expressed as fold change over the WT BMDM. Mean ± SEM, *n* = 4. **b** RNA Pol II occupancy at the *Atp1b3* gene. **c** (Left) Nascent *Atp1b3* pre-mRNA levels from WT and *Trim33*^−/−^ BMDM treated with DMSO or Flavopiridol (FLAVO) for 4 h, expressed as fold change over the DMSO-treated WT BMDM. RT-qPCR were performed using total RNA and intronic positions of primers are relative to TSS. (Right) Fold decrease, expressed as the ratio between DMSO and FLAVO-treated BMDM. Mean ± SEM, *n* = 5. **d** Antisense promoter transcripts levels (450nt upstream the TSS) in WT and *Trim33*^−/−^ BMDM, relative to expression of WT BMDM. Mean ± SEM, *n* = 5. **e** ChIP-seq profiles of indicated chromatin modifications along with ChromHMM analysis at the *Atp1b3* gene. **f** SPT16 ChIP-seq profiles at the *Atp1b3* locus. **g** (Up) Schematic for EcoRI fragments and PCR primers positions used in Chromosome Conformation Capture (3C) at the *Atp1b3*/*Rnf7* locus. (Down) 3C analysis of interaction between the − 35 kb region and the *Atp1b3* promoter (− 35 kb/ *Atp1b3* prom) in WT and *Trim33*^−/−^ BMDM (two independent replicates). Negative controls (− 35 kb/− 92 kb and *Atp1b3* prom/− 92 kb) included a region located 92 kb upstream the *Atp1b3* TSS and not bound by TRIM33. Positive controls (Control) included digested and ligated genomic PCR fragments containing all ligation junctions. Genomic DNA after ligation (Input) is shown as a loading control. **h** (Left) RNA Pol II ChIP-qPCR at the *Atp1b3* promoter 4 h after treatment of WT and *Trim33*^−/−^ BMDM with the FACT inhibitor CBL0137 (CBL). (Right) Fold decrease, expressed as the ratio between DMSO and CBL-treated BMDM. Mean ± SEM, *n* = 3. **i** (Left) *Atp1b3* pre-mRNA levels in WT and *Trim33*^−/−^ BMDM 4 h after CBL treatment, relative to expression of DMSO-treated WT BMDM. RT-qPCR was performed using total RNA. (Right) Fold decrease, expressed as the ratio between DMSO and CBL-treated BMDM. Mean ± SEM, *n* = 3

In *Trim33*^−/−^ BMDM, loss of FACT recruitment at the − 35 kb site correlated with increased mRNA levels of the nearby genes *Atp1b3* and *Rnf7* (Fig. 3a). Increased mRNA levels were also observed for other genes associated with regions where SPT16 binding is decreased in *Trim33*^−/−^ BMDM (Additional file 3: Figure S3a), suggesting a repressive role of the FACT/TRIM33 complex in BMDM. In accordance, compared to WT BMDM, *Trim33*^−/−^ BMDM displayed a higher density of RNA Pol II into *Atp1b3* gene body and at the 3′ end but not at the TSS (Fig. 3b). Similar results were found for the *Rnf7* gene (Additional file 3: Figure S3b). Quantification of nascent transcripts of the *Atp1b3* gene in WT and *Trim33*^−/−^ BMDM showed increased level of *Atp1b3* pre-mRNA in *Trim33*^−/−^ BMDM (Fig. 3c). This increased level was not associated with an increased transcription elongation as treatment with flavopiridol, a specific inhibitor of the positive transcription elongation factor P-TEFb [30], resulted in similar fold decrease of nascent transcripts in WT and *Trim33*^−/−^ BMDM (Fig. 3c). Antisense promoter transcription, a mark of high transcriptional activity [31], was increased in *Trim33*^−/−^ BMDM (Fig. 3d) and integration of three chromatin datasets by ChromHMM in *Atp1b3* gene body showed broader H3K4me3 and H3K79me2 but not H3K36me3 regions in *Trim33*^−/−^ BMDM compared to WT BMDM (Fig. 3e) indicating that increased transcription of the *Atp1b3* gene in *Trim33*^−/−^ BMDM was mainly accounted for by enhanced transcription initiation rather than transcription elongation. Similar results were found for the *Rnf7* gene (Additional file 3: Figure S3c). These results suggest that TRIM33 may act as a transcriptional switch for the *Atp1b3* gene in macrophages. In accordance, serum deprivation of the monocyte/macrophage RAW 264.7 (RAW) cell line, a stress condition that mimics lack of nutrients, resulted in decreased TRIM33 protein expression (Additional file 3: Figure S3d) with a concomitant loss of TRIM33 occupancy at the − 35 kb site (Additional file 3: Figure S3e) and increased levels of *Atp1b3* mRNA (Additional file 3: Figure S3f) and protein (Additional file 3: Figure S3g).

In the *Atp1b3* gene body, SPT16 and SSRP1 occupancy was higher in *Trim33*^−/−^ BMDM compared to WT BMDM (Fig. 3f and Additional file 4: Figure S4a). Since FACT did not bind at the − 35 kb site in *Trim33*^−/−^ BMDM, it was tempting to speculate that TRIM33 could sequester the FACT complex at the − 35 kb region to limit *Atp1b3* transcription. We first showed that the − 35 kb region was in spatial proximity with the promoter of the *Atp1b3* gene in WT and *Trim33*^−/−^ BMDM (Fig. 3g). Similar results were found for the *Rnf7* promoter (Additional file 4: Figure S4b). Second, we studied the effect of FACT inhibition on the transcription of the *Atp1b3* gene, using the small molecule CBL0137, which can induce FACT-mediated chromatin disassembly without any DNA damage [32, 33]. CBL0137 has also a FACT-independent effect in several mouse tissues and fibroblast cells by activating the interferon response [34]. Analyses of mRNA levels of IFN-responsive genes, *Irf7*, *Ifit3* and *Isg15,* showed that CBL0137 did not activate interferon response in WT and *Trim33*^−/−^ BMDM (Additional file 4: Figure S4c), indicating that the effect of CBL0137 is not mediated by the interferon response in BMDM. At the *Atp1b3* locus, CBL0137 treatment did not abrogate the chromatin loop between the − 35 kb region and the *Atp1b3* or *Rnf7* promoter (Additional file 4: Figure S4d). RNA Pol II occupancy at the promoter of the *Atp1b3* gene was similar in WT and *Trim33*^−/−^ BMDM after CBL0137 treatment, whereas it was 2.5-fold higher in *Trim33*^−/−^ BMDM than in WT BMDM without treatment (Fig. 3h). Furthermore, quantification of nascent transcripts in WT and *Trim33*^−/−^ BMDM treated with CBL0137 showed a tenfold decreased level of *Atp1b3* pre-mRNA in *Trim33*^−/−^ versus WT BMDM resulting in similar level of nascent transcripts in *Trim33*^−/−^ and WT BMDM 4 h after CBL0137 treatment (Fig. 3i).

Similar results were found for other genes potentially regulated by a distal regulatory sequence bound by a SPT16/TRIM33 complex without any TRIM33 or PU.1 binding at their promoter. In *Trim33*^−/−^ BMDM, decreased SPT16 occupancy at distal regulatory regions was associated with an increased SPT16 binding at the nearest TSS and/or gene body, enhanced RNA Pol II occupancy (Additional file 4: Figure S4e) and increased mRNA expression (Additional file 3: Figure S3a). In accordance, CBL0137 treatment resulted in a decreased transcription in absence of TRIM33 (Additional file 4: Figure S4f).

Altogether, these results show that RNA Pol II recruitment at the *Atp1b3* promoter was FACT-dependent, that TRIM33 represses transcription of the *Atp1b3* gene by sequestering the FACT complex at the − 35 kb region and suggest a repressive role of the SPT16/TRIM33 complex at distal regulatory regions in macrophages.

### FACT/TRIM33 complex regulates nucleosome dynamics at the − 35 kb regulatory region

**Fig. 4:**
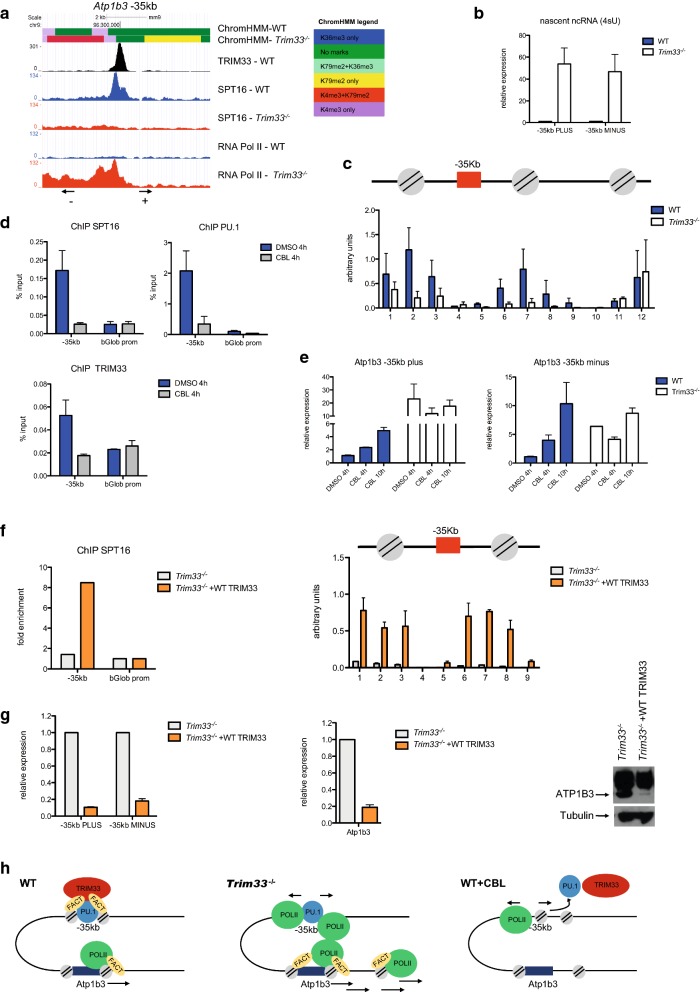
Nucleosome dynamics and non-coding RNA production at the − 35 kb regulatory region. **a** TRIM33, SPT16 and RNA Pol II occupancy along with ChromHMM analysis over the *Atp1b3* − 35 kb site. **b** Nascent bidirectional transcripts at the − 35 kb site in WT and *Trim33*^−/−^ BMDM, measured after 4sU-tagging and purification of newly transcribed RNA. Data are relative to WT BMDM. Mean ± SEM, *n* = 3. **c** Nucleosome positioning at the − 35 kb region in MNase-digested chromatin from WT and *Trim33*^−/−^ BMDM. Data are relative to the undigested chromatin. Mean ± SEM, *n* = 3. **d** ChIP-qPCR of SPT16, PU.1 and TRIM33 occupancy at the − 35 kb site in WT BMDM treated with DMSO or CBL for 4 h. Mean ± SEM, *n* = 3. **e** Kinetics of non-coding transcripts at the − 35 kb region in WT and *Trim33*^−/−^ BMDM after CBL treatment. RT-qPCR was performed using total RNA, and data are presented relative to expression of DMSO-treated WT BMDM. Mean ± SEM, *n* = 3. **f** SPT16 ChIP-qPCR (left) and nucleosome positioning (right) at the − 35 kb region in *Trim33*^−/−^ IM (*Trim33*^−/−^) and in *Trim33*^−/−^ IM that contained a lentivirus expressing TRIM33 (*Trim33*^−/−^ + WT TRIM33). Mean ± SEM, *n* = 3. **g** Non-coding transcripts at the − 35 kb region (left), *Atp1b3* mRNA (middle) and protein expression (right) in *Trim33*^−/−^ and *Trim33*^−/−^ + WT TRIM33 IM. Data are presented relative to expression of *Trim33*^−/−^ IM. Mean ± SEM, *n* = 4. **h** Model for FACT/TRIM33-mediated repression at the *Atp1b3* locus

In WT BMDM, the − 35 kb region contained neither H3K4me1 nor H3K4me2 enhancer marks but displayed H3K27ac and H3K4me3 marks (Fig. 4a and Additional file 5: Figure S5a). In *Trim33*^−/−^ BMDM, the − 35 kb region did not acquire H3K4me1 enhancer mark (Additional file 5: Figure S5b) but displayed marks of active transcription with a H3K4me3-marked region flanked by H3K79me2 and recruitment of RNA Pol II at the highest SPT16 peak (Fig. 4a). In accordance, the − 35 kb site produced bidirectional transcripts in *Trim33*^−/−^ BMDM but was not transcribed in WT BMDM (Fig. 4b). Position of nucleosomes in the − 35 kb region was next investigated after MNase digestion on cross-linked chromatin. No global difference in chromatin sensitivity to MNase digestion was observed in WT and *Trim33*^−/−^ BMDM (Additional file 5: Figure S5c). Cross-linked chromatin was thus digested with MNase to obtain mononucleosomes (Additional file 5: Figure S5d) and qPCR were performed on purified DNA using overlapping amplicons. Two positioned nucleosomes surrounding the − 35 kb TRIM33/PU.1 peak and corresponding to the FACT binding sites were found in WT BMDM but not in *Trim33*^−/−^ BMDM (Fig. 4c). Upon inactivation of FACT by CBL0137 treatment of WT BMDM, both PU.1 and TRIM33 binding at the − 35 kb region were abolished (Fig. 4d), probably as a consequence of extensive chromatin disassembly induced by FACT inhibition. Importantly, bidirectional transcripts at the − 35 kb region were detected in WT BMDM upon CBL0137 treatment (Fig. 4e), strengthening the idea that an interplay between FACT, PU.1 and TRIM33 is critical to maintain a chromatin structure that represses local non-coding transcription at the − 35 kb. Exogenous expression of TRIM33 in *Trim33*^−/−^ immortalized macrophages (IM) restored TRIM33 (Additional file 5: Figure S5e) and SPT16 occupancy at the − 35 kb region (Fig. 4f, left panel). The SPT16/TRIM33 binding was associated with reconstitution of nucleosomes positioning at the − 35 kb site (Fig. 4f, right panel), repression of bidirectional transcription at the − 35 kb site (Fig. 4g, left panel) and decreased mRNA (Fig. 4g, middle panel) and protein levels of *Atp1b3* (Fig. 4g, right panel).

## Discussion

The roles of FACT in transcription elongation and in nucleosomes organization are well characterized [35]. However, any role of FACT in the dynamic remodeling of chromatin at distal regulatory regions has not been shown. Here, we describe a novel genome-wide profile in which FACT subunit SPT16 binds on positioned nucleosomes, flanking PU.1-occupied sites in proximity of genes essential for macrophage development and function. In mouse embryonic stem cells, FACT can interact with the transcription factor Oct4 [36, 37] and have a role on cell fate determination [38]. FACT can also safeguard cell identities and acts as a barrier for cell fate reprogramming in human fibroblasts [39]. Our results suggest a potential role of FACT subunit SPT16 in shaping chromatin landscape at distal gene regulatory regions to establish and/or maintain PU.1 binding and macrophage identity. Given the role of PU.1 in priming distal regulatory sequences for stimuli-dependent activation, it remains to be determined if SPT16 could also be involved in chromatin organization changes that occur at distal regulatory regions upon stimulation of macrophages.

Intergenic SPT16-occupied regions displayed an active chromatin signature (high H3K27ac and H3K4me3, but low H3K4me1) and RNA Pol II binding and were found near actively transcribed genes, suggesting a potential contribution of intergenic SPT16 binding in maintaining and/or activating expression of genes regulated by PU.1. Addressing a specific role of intergenic SPT16 in nearby gene transcription is challenging as inhibition of FACT causes impaired transcription elongation of nearby genes as a direct consequence of FACT release from their gene bodies.

In a subset of these macrophage distal regulatory regions, SPT16 can interact with TRIM33 and TRIM33 deficiency leads to decrease of SPT16 binding along with increased chromatin accessibility. At several distal regulatory regions, these changes are associated with increased SPT16 and RNA Pol II occupancy at nearby genes, suggesting that sequestering of SPT16 by TRIM33 might limit nearby gene transcription. FACT has been previously identified as interacting partner of TRIM33 in erythroid cells, where the FACT/TRIM33 complex is necessary for efficient transcription elongation of erythroid genes [7]. In macrophages, TRIM33 participates to FACT recruitment at gene distal regulatory regions and the FACT/TRIM33 complex may act as a repressor further reinforcing the idea of cell type-specific functions conferred by FACT. Repression at distal regulatory elements in macrophages is presently accounted for by inhibition of histone acetylation through recruitment of the NuRD and the NCOR/SMRT multiprotein co-repressor complexes by various transcription factors or by impairing recruitment of co-activators such as CBP/p300 [40–43]. Using *Atp1b3* gene as a model, we identified a regulatory region, bound by the FACT/TRIM33 complex, that is critical to maintain a chromatin structure that represses local non-coding transcription and limits the binding of FACT on the *Atp1b3* promoter (Fig. 4h, left panel). TRIM33 deficiency leads to FACT release from this region, disassembly of two positioned nucleosomes, acquisition of histone marks of active transcription, RNA Pol II recruitment and non-coding RNA production together with increased recruitment of FACT at the *Atp1b3* promoter and increased *Atp1b3* gene transcription (Fig. 4h, middle panel). Treatment of WT BMDM with a specific FACT inhibitor supports the role of FACT in stabilizing positioning of nucleosomes on this regulatory region (Fig. 4h, right panel). Other transcription elongation factors, such as Spt6 or Ell3, have been recently shown to bind super-enhancers or enhancers where they control the balance of H3K27 acetylation and methylation and enhancer transcription [44] or are required for priming developmentally regulated genes for transcriptional activation during differentiation [45]. Altogether, these results indicate that elongation factors might also have a role in the transcriptional activity of distal regulatory regions.

Our results also indicate that TRIM33 might act as a transcriptional switch for the *Atp1b3* gene in macrophages as, upon serum deprivation, TRIM33 protein levels rapidly decrease with a concomitant loss of TRIM33 occupancy at − 35 kb site and rapid induction of *Atp1b3* mRNA and protein levels. The *Atp1b3* gene encodes the β3 subunit of the Na, K ATPase, known to maintain the electrochemical gradients of sodium and potassium across the plasma membrane. Even if the biological relevance of increased ATP1B3 levels in BMDM upon serum deprivation is unknown, ATP1B3 has been shown to interfere with viral infections in human cells. Enterovirus infection can induce increased ATP1B3 levels that regulate the immune response by inducing the production of type I IFNs in RD muscle cell line [46] and ATP1B3 protein can modulate HIV-1 restriction and NF-κB activation in a BST-2-dependent manner in Hela cells [47]. By mimicking the effects of viral infection (by treatment with the pIpC, a TLR3 agonist, or by Ifnb1 treatment) or other stimulation (by treatment with LPS, a TLR4 agonist), we did not find any activation of the *Atp1b3* gene in both WT and *Trim33*^−/−^ BMDM (Additional file 5: Figure S5f), suggesting that ATP1B3 functions could be restricted to specific viral infections. However, as TRIM proteins are involved in the immune response to infections and TRIM33 directly regulates IFNβ levels in activated macrophages, it could be interesting to speculate if TRIM33 might also be a transcriptional switch that activates genes involved in the immune response to virus in macrophages.

## Conclusion

Whereas further investigation will be required to determine the role of FACT in the chromatin landscape of macrophages, our results provide new mechanistic insights into the interplay between SPT16 and TRIM33 in remodeling chromatin at distal regulatory elements and in transcription regulation in macrophages.

## Methods

### Mice, cell culture and treatments

To generate deletion of *Trim33* in mature myeloid cells, *Trim33*^*fl*/*fl*^ C57Bl/6-CD45.2 mice [5] were crossed with Lysozyme-Cre C57Bl/6-CD45.2 mice (strain name: B6.129P2-Lyz2tm1(cre)Ifo/J, The Jackson Laboratory). WT and *Trim33*^−/−^ BMDM and immortalized macrophages (IM) were obtained as previously described [9]. BMDM were differentiated for 7 days in IMDM supplemented with 10% FCS and 25 ng/ml M-CSF (Miltenyi Biotec). IM were cultured in IMDM supplemented with 20% FCS and 50 ng/ml M-CSF. RAW 264.7 (ATCC) cells were cultured in DMEM supplemented with 10% FCS. Flavopiridol (Sigma Aldrich) was used at 1 µM, CBL0137 (Cayman Chemical) at 3 µM.

### Chromatin Immunoprecipitation (ChIP) and ChIP-seq analyses

ChIP experiments were performed essentially as previously described [9]. For ChIP-seq, libraries were prepared with the MicroPlex Library Preparation kit v2 (Diagenode) and sequenced on Illumina Hiseq 4000 following Illumina’s instructions.

Single-end 50 bp reads were mapped to the mm9 genome assembly using Bowtie 1.0.0 with the following arguments: -m 1 –strata –best -y -S -l 40 -p 2. Peak detection was performed using the MACS software [48] with the corresponding input as control sample. Regions were considered significantly enriched at a *p* value threshold of 1e−05. Peaks were then annotated according to genomic features of Ensembl v67 using Homer (http://homer.salk.edu/homer/). Promoter-TSS was defined from − 1 kb to + 100 bp relative to the position of the TSS and TTS from − 100 bp to + 1 kb relative to the position of the TTS. Intergenic SPT16 peaks that overlap with the mm9 blacklist from ENCODE were additional filtered out. Functional annotation analyses were performed using GREAT or DAVID web-tools. Overlap between SPT16 and PU.1 or TRIM33 peaks was performed using BedTools, requiring at least 1 bp overlap. Enrichment profiles and comparisons of ChIP-seq datasets at indicated regions were performed using DeepTools on Galaxy (https://usegalaxy.org/). Data were represented as heatmaps, average density plots or boxplots. Annotation of combinatorial chromatin states for H3K4me3, H3K79me2 and H3K36me3 marks was performed with the ChromHMM software (http://compbio.mit.edu/ChromHMM/) at 200 bp bin interval in WT and *Trim33*^−/−^ BMDM using the corresponding input as background control.

### ATAC-seq

ATAC-seq was carried out as previously described [49], in two biological replicates. Nuclei were isolated from 50.000 BMDM with lysis buffer (10 mM Tris-HCl, 10 mM NaCl, 3 mM MgCl_2_, 0.1% Igepal) and resuspended in Tn5 transposase mixture (Nextera Tn5 Transposase, 1xTagment DNA buffer (Illumina), and 0.2 mg/ml digitonin (Promega). The tagmentation reaction was carried out for 30 min at 37 °C followed by DNA purification using the MinElute Reaction Cleanup kit (Qiagen). Tagmented DNA was amplified with NEBNext High-Fidelity PCR Master Mix (NEB) and sequencing primers Ad1_noMX and Ad2.1-4 indexing primers [49]. Libraries were cleaned and size-selected using Spriselect beads (Beckman) and sequenced on Illumina Hiseq 4000. Paired-end 100 bp reads were mapped to the mm9 genome assembly using Bowtie v1.0.0 with default parameters except for “-p 3 -m 1 -X2000” and filtered to remove duplicated reads, reads with mapping quality < 10, reads from ENCODE blacklisted regions and mitochondrial reads. Normalized BigWig files were generated using Homer makeUCSCfile with the following parameter “-norm 4e7 -style dnase.” Differentially accessible regions were identified by DESeq2.

### Nucleosome positioning

Cells were cross-linked with 1% formaldehyde and lysed in PBS/0.5% Triton X-100. Micrococcal nuclease (MNase) digestion was performed on isolated nuclei with indicated concentrations of MNase (New England Biolabs) in 50 mM Tris-HCl, 5 mM CaCl_2_ for 10 min at 37 °C. Reaction was stopped by addition of excess of EDTA. The nuclei were then digested by 0.5 mg/ml of Proteinase K (Invitrogen) and DNA isolated by phenol-chloroform extraction. Purified DNA was then run in a 1% agarose gel and the mononucleosomal band cut and purified with the Qiagen gel extraction kit. The resulting material was analyzed by sets of overlapping primer pairs, each generating amplicons of 60–80 bp with 15–25 bp overlap between neighboring amplicons.

### 4sU-tagging

For metabolic labeling of newly synthesized RNA, BMDM were incubated with 150 μM of 4sU (4-thiouridine, Sigma Aldrich) for 10 min prior to RNA extraction. Total RNA was extracted with the miRNeasy kit (Qiagen). Biotinylation reaction was carried out with 1 µg of total RNA in labeling buffer (10 mM Tris pH 7.4, 1 mM EDTA) and 0.2 mg/ml EZ-Link HPDP-Biotin (Pierce) for 2 h at room temperature. Unbound HPDP-Biotin was removed by chloroform/isoamylalcohol (24:1) extraction. Biotinylated RNA was isolated using streptavidin magnetic beads (New England Biolabs), eluted in 100 mM DTT and cleaned up with RNeasy MinElute Spin columns (Qiagen).

### RNA extraction and RT-qPCR

Total RNA was extracted with the RNeasy Plus Micro kit (Qiagen) and reverse transcribed with random primers and Superscript IV (Life Technologies). Quantitative PCR was performed using the Power SYBR green PCR master mix (Applied Biosystems).

### Chromosome conformation capture (3C)

3C assays were carried out essentially as described previously [50]. Briefly, cells were fixed with 2% formaldehyde and lysed in 10 mM Tris pH8, 10 mM NaCl, 0.2% NP-40. Nuclei were resuspended in EcoRI buffer (New England Biolabs) containing 0.3% SDS for 1 h at 37 °C. After addition of 2% Triton X-100 for 1 h at 37 °C, nuclei were digested overnight with 400U of EcoRI-HF (New England Biolabs). Samples were incubated with 1.6% SDS at 65 °C, diluted tenfold with 1.15× ligase buffer (Roche) and incubated with 1% Triton X-100 at 37 °C. Ligation was performed for 4 h at 16 °C with 100U of T4 DNA ligase (Roche). After de-cross-linking and RNA removal, DNA was purified and analyzed by PCR using unidirectional primers designed to amplify across ligation junctions. Positive control PCR were carried out using genomic PCR fragments covering the restriction sites of interest that were then digested and ligated.

### Protein extraction, immunoprecipitation and mass spectrometry

For TRIM33 and ATP1B3 immunoblotting, total proteins were extracted in lysis buffer (25 mM Hepes, 100 mM NaCl, 5 mM MgCl_2_, 10% glycerol, 0.2% NP-40 and protease inhibitor cocktail).

For mass spectrometry, IM were resuspended in hypotonic buffer (10 mM HEPES, 1.5 mM MgCl_2_, 10 mM KCl and protease inhibitor cocktail) and nuclei were extracted in 20 mM HEPES-KOH pH 7.9, 25% glycerol, 420 mM NaCl, 1.5 mM MgCl_2_, 0.2 mM EDTA, 0.5 mM DTT and protease inhibitor cocktail. Immunoprecipitation of FLAG-TRIM33 was carried out with 10ug of FLAG antibody cross-linked to protein G-Sepharose beads (Millipore), as previously described [51]. In-gel enzymatic digestion and mass spectrometry analysis were carried out as previously described [52]. Proteins with no peptides identified in the control experiment from *Trim33*^−/−^ IM were considered as positive hits.

### Antibodies and primers

The following antibodies were used in immunoblotting, immunprecipitation and ChIP:

TRIM33 (3E9, Euromedex for WB and IP; A301-059A, Bethyl for ChIP), SPT16 (sc-28734, Santa Cruz), SSRP1 (10D1, Biolegend), PU.1 (sc-352X, Santa Cruz), ATP1B3 (ab137055, Abcam), RNA POLII (sc-9001X, Santa Cruz), H3K4me3 (07-473, Millipore), H3K79me2 (ab3594, Abcam), H3K36me3 (ab9050, Abcam), FLAG (M2 monoclonal, Sigma Aldrich).

Primer sequences for RT-qPCR are available upon request.

## Additional files

**Additional file 1: Figure S1.** **Related to Fig.** 1**. a** Examples of SPT16 ChIP-seq profiles in BMDM along with RNA Pol II occupancy. **b** Functional annotations of genes nearest to intergenic SPT16 peaks in BMDM. **c** SSRP1 ChIP-qPCR analysis at indicated SPT16-bound distal regulatory regions in BMDM. A region in the beta globin promoter is used as a negative control. **d** Representative examples of SPT16 ChIP-seq profiles in BMDM along with RNA Pol II occupancy and ChromHMM analysis at indicated genes associated with intergenic SPT16 peaks

**Additional file 2: Figure S2.** **Related to Fig.** 2**. a** Overlap between SPT16 and TRIM33 peaks in BMDM. **b** Gene Ontology (GO) annotations of genes nearest to intergenic SPT16/TRIM33 peaks in BMDM. **c** Scatter plot showing global correlation of SPT16 binding in WT and *Trim33*^−/−^ BMDM. The Spearman coefficient value is given. **d** Normalized SPT16 tag count at most enriched (Top10%) intergenic SPT16 peaks that did not colocalized with TRIM33. ns: not significant, Paired *t* test. **e** SSRP1 ChIP-qPCR at indicated SPT16/TRIM33 bound regions in WT and *Trim33*^−/−^ BMDM. Mean ± SEM, *n* = 3. **f** PU.1 ChIP-qPCR analysis at indicated SPT16/TRIM33 bound regions in WT and *Trim33*^−/−^ BMDM. Mean ± SEM, *n* = 3. **g** SPT16 ChIP-qPCR in WT and *Trim33*^−/−^ BMDM (left) and in *Trim33*^−/−^ immortalized macrophages (IM) and in *Trim33*^−/−^ IM rescued with exogenous TRIM33 (*Trim33*^−/−^ + WT TRIM33) (right). **h** Immunoblotting of FLAG-TRIM33, PU.1 and b-ACTIN in Hela cells transfected with the indicated vectors (left). FLAG-TRIM33 ChIP-qPCR at the reporter vector containing three PU.1 binding sites. Mean ± SEM, *n* = 2. **i** PU.1 and TRIM33 occupancy at the *Atp1b3*/*Rnf7* locus in BMDM

**Additional file 3: Figure S3.** **Related to Fig.** 3**. a** mRNA levels of *Tgfbi*, *Mmp12*, *Cd72* and *Napepld* in WT and *Trim33*^−/−^ BMDM. Data are presented relative to expression of WT BMDM. Mean ± SEM, *n* = 4. **b** UCSC genome browser image of RNA Pol II at the *Rnf7* gene in WT and *Trim33*^−/−^ BMDM. **c** UCSC genome browser image of indicated chromatin modifications along with ChromHMM analysis at the *Rnf7* gene in WT and *Trim33*^−/−^ BMDM. **d** Immunoblotting showing TRIM33 expression in RAW cells grown in culture medium containing 10% or 0.1% FCS for 2 h and 6 h. **e** TRIM33 occupancy at the − 35 kb site in RAW cells grown in culture medium containing 10% or 0.1% FCS for 2 h and 6 h. Mean ± SEM, *n* = 2. **f** *Atp1b3* mRNA expression levels in RAW cells grown in culture medium containing 10% or 0.1% FCS for 2 h and 6 h. Mean ± SEM, *n* = 2. **g** Immunoblotting showing ATP1B3 expression in RAW cells grown in culture medium containing 10% or 0.1% FCS for 2 h and 6 h

**Additional file 4: Figure S4.** **Related to Fig.** 3**. a** SSRP1 ChIP-qPCR at the indicated positions in the *Atp1b3* gene in WT and *Trim33*^−/−^ BMDM. **b** 3C analyses of interaction between the − 35 kb region and the *Rnf7* promoter. See Fig. 3g for the schematic of primers positions. **c** Kinetics of *Irf7*, *Ifit3* and *Isg15* mRNA relative expression in WT and *Trim33*^−/−^ BMDM treated with CBL. Data are presented relative to expression of untreated WT BMDM. Mean ± SEM, *n* = 3. **d** 3C analyses at the *Atp1b3*/*Rnf7* locus in WT and *Trim33*^−/−^ BMDM treated for 4 h with CBL. See Fig. 3g for the schematic of primers positions. **e** SPT16 and RNA Pol II metaprofile analyses at indicated gene bodies in WT and *Trim33*^−/−^ BMDM. **f** Fold decrease in WT and *Trim33*^−/−^ BMDM, expressed as the ratio between DMSO and CBL-treated BMDM, of pre-mRNA levels of genes regulated by an intergenic SPT16/TRIM33 complex. Mean ± SEM, *n* = 3

**Additional file 5: Figure S5.** **Related to Fig.** 4**. a** H3K4me1, H3K4me2 and H3K27ac histone modification marks at the − 35 Kb region in BMDM. Also shown the TRIM33 peak. **b** H3K4me1 ChIP-qPCR at the − 35 kb region in WT and *Trim33*^−/−^ BMDM. Regions near to *Ifnb1* and *Irf1* genes are used as positive controls and a region in the beta globin promoter is used as a negative control. **c** Representative image of MNase digestion profile. Nuclei from WT and *Trim33*^−/−^ BMDM were digested with increasing amounts of MNase. **d** Representative image of MNase digestion profiles of chromatin from WT and *Trim33*^−/−^ BMDM before mono-nucleosome purification. **e** TRIM33 ChIP-qPCR at the − 35 kb region in *Trim33*^−/−^ and *Trim33*^−/−^ + WT TRIM33 IM. **f** Kinetics of *Atp1b3* mRNA levels in WT and *Trim33*^−/−^ BMDM treated for the indicated times with pIpC (left), Ifnb1 (middle) and LPS (right). Data are presented relative to expression of untreated WT BMDM

## Data Availability

The datasets generated during the current study are available in the Gene Expression Omnibus (GEO) repository under accession number GSE132924. H3K27ac and H3K4me1 ChIP-seq datasets were retrieved from ENCODE, MNase-seq from GSE50762, PU.1 ChIP-seq from GSE19553, H3K4me3 and TRIM33 ChIP-seq datasets from GSE43654.

## Abbreviations

FACT: facilitates chromatin transcription
BMDM: bone marrow-derived macrophages
MNase: micrococcal nuclease
ATAC: Assay for Transposase Accessible Chromatin
TSS: transcription start site
IM: immortalized macrophages
