# Supplementary material for: Interplay between FACT subunit SPT16 and TRIM33 can remodel chromatin at macrophage distal regulatory elements
Source: Epigenetics Chromatin. 2019 Jul 22;12:46. doi: 10.1186/s13072-019-0288-3 (PMC6647326; doi:10.1186/s13072-019-0288-3)
Supplement: Supplementary file 1 — Additional file 1: Figure S1. Related to Fig. 1. a Examples of SPT16 ChIP-seq profiles in BMDM along with RNA Pol II occupancy. b Functional annotations of genes nearest to intergenic SPT16 peaks in BMDM. c SSRP1 ChIP-qPCR analysis at indicated SPT16-bound distal regulatory regions in BMDM. A region in the beta globin promoter is used as a negative control. d Representative examples of SPT16 ChIP-seq profiles in BMDM along with RNA Pol II occupancy and ChromHMM analysis at indicated genes associated with intergenic SPT16 peaks [file 13072_2019_288_MOESM1_ESM.pdf]

a

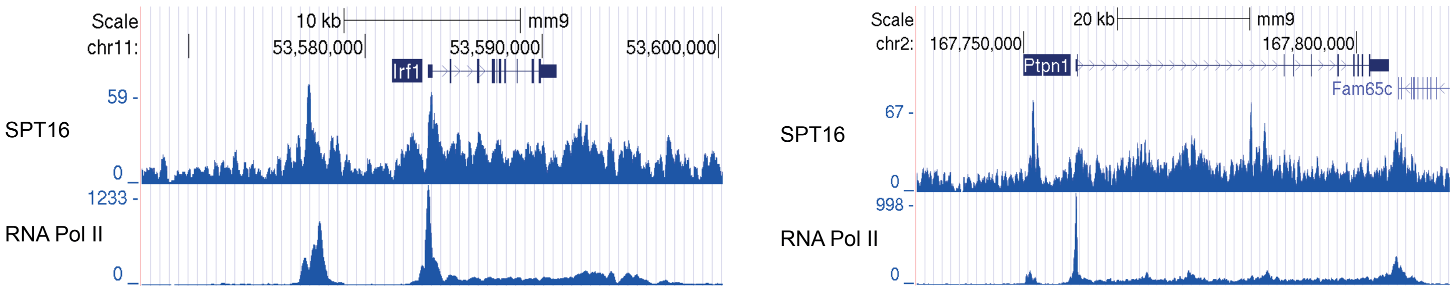

b

Annotation	Description	Count	P_Value
UP_KEYWORDS	Transcription regulation	146	7.8E-12
UP_KEYWORDS	Transcription	149	1.2E-11
GOTERM_BP	transcription, DNA-templated	153	5.0E-9
UP_KEYWORDS	Immunity	47	9.7E-9
GOTERM_BP	immune system process	46	1.6E-7
UP_KEYWORDS	Innate immunity	28	1.7E-5

c

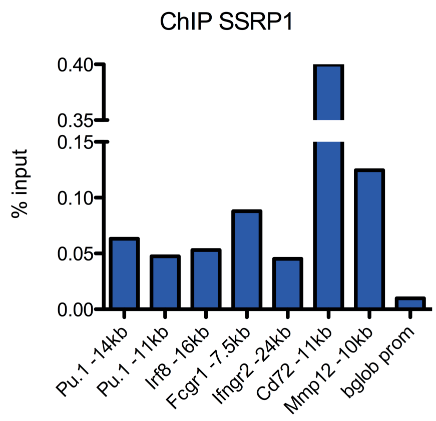

d

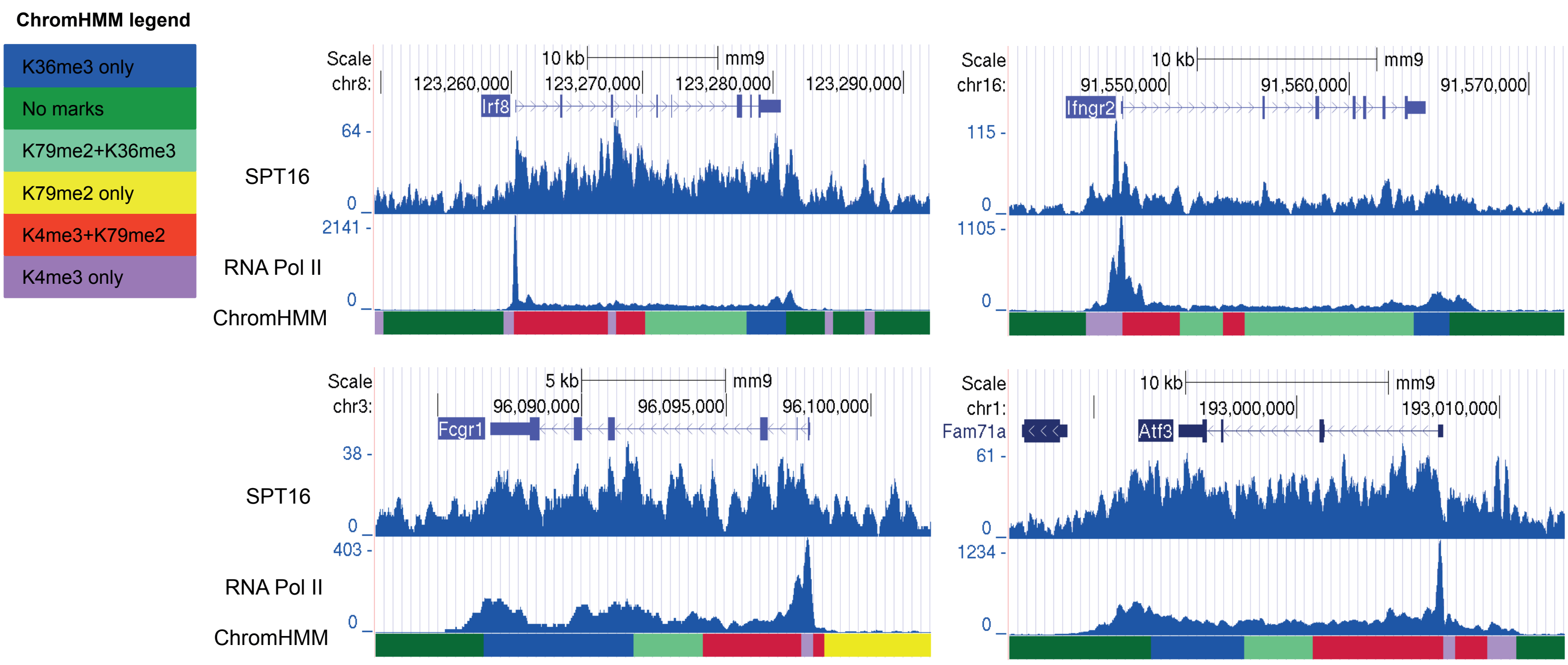

FigureS1
