# Supplementary material for: Interplay between FACT subunit SPT16 and TRIM33 can remodel chromatin at macrophage distal regulatory elements
Source: Epigenetics Chromatin. 2019 Jul 22;12:46. doi: 10.1186/s13072-019-0288-3 (PMC6647326; doi:10.1186/s13072-019-0288-3)
Supplement: Supplementary file 2 — Additional file 2: Figure S2. Related to Fig. 2. a Overlap between SPT16 and TRIM33 peaks in BMDM. b Gene Ontology (GO) annotations of genes nearest to intergenic SPT16/TRIM33 peaks in BMDM. c Scatter plot showing global correlation of SPT16 binding in WT and Trim33−/− BMDM. The Spearman coefficient value is given. d Normalized SPT16 tag count at most enriched (Top10%) intergenic SPT16 peaks that did not colocalized with TRIM33. ns: not significant, Paired t test. e SSRP1 ChIP-qPCR at indicated SPT16/TRIM33 bound regions in WT and Trim33−/− BMDM. Mean ± SEM, n = 3. f PU.1 ChIP-qPCR analysis at indicated SPT16/TRIM33 bound regions in WT and Trim33−/− BMDM. Mean ± SEM, n = 3. g SPT16 ChIP-qPCR in WT and Trim33−/− BMDM (left) and in Trim33−/− immortalized macrophages (IM) and in Trim33−/− IM rescued with exogenous TRIM33 (Trim33−/− + WT TRIM33) (right). h Immunoblotting of FLAG-TRIM33, PU.1 and b-ACTIN in Hela cells transfected with the indicated vectors (left). FLAG-TRIM33 ChIP-qPCR at the reporter vector containing three PU.1 binding sites. Mean ± SEM, n = 2. i PU.1 and TRIM33 occupancy at the Atp1b3/Rnf7 locus in BMDM [file 13072_2019_288_MOESM2_ESM.pdf]

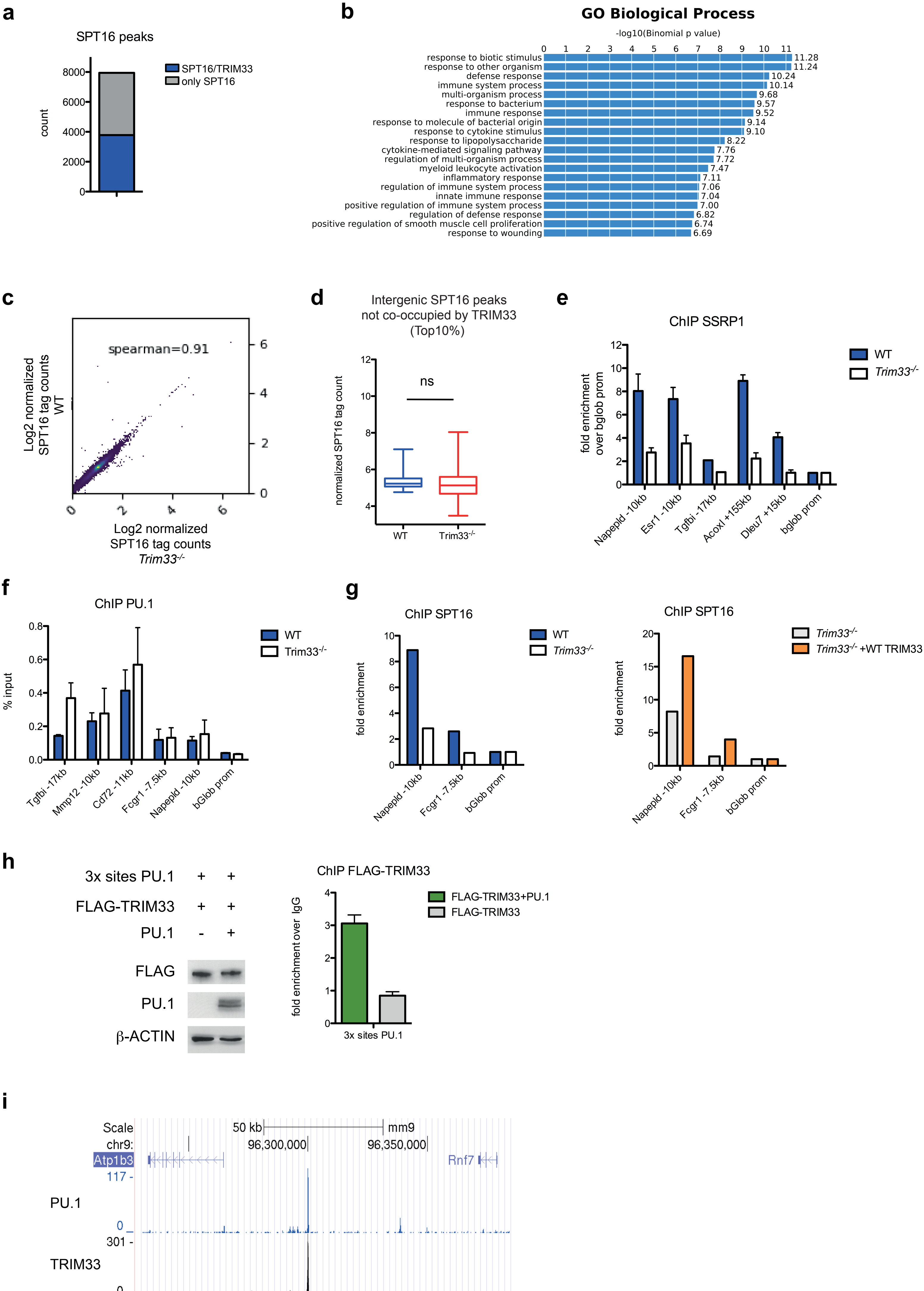
a
SPT16 peaks
count
8000
6000
4000
2000
0
SPT16/TRIM33
only SPT16
b
GO Biological Process
-log10(Binomial p value)
0 1 2 3 4 5 6 7 8 9 10 11
response to biotic stimulus 11.28
response to other organism 11.24
defense response 10.24
immune system process 10.14
multi-organism process 9.68
response to bacterium 9.57
immune response 9.52
response to molecule of bacterial origin 9.14
response to cytokine stimulus 9.10
response to lipopolysaccharide 8.22
cytokine-mediated signaling pathway 7.76
regulation of multi-organism process 7.72
myeloid leukocyte activation 7.47
inflammatory response 7.11
regulation of immune system process 7.06
innate immune response 7.04
positive regulation of immune system process 7.00
regulation of defense response 6.82
positive regulation of smooth muscle cell proliferation 6.74
response to wounding 6.69
c
spearman=0.91
Log2 normalized SPT16 tag counts WT
Log2 normalized SPT16 tag counts Trim33-/-
0 2 4 6
d
Intergenic SPT16 peaks not co-occupied by TRIM33 (Top10%)
normalized SPT16 tag count
ns
WT
Trim33-/-
e
ChIP SSRP1
fold enrichment over bglob prom
WT
Trim33-/-
Napepld -10kb
Esr1 -10kb
Tgfbi -17kb
Acoxl +155kb
Dleu7 +15kb
bglob prom
f
ChIP PU.1
% input
WT
Trim33-/-
Tgfbi -17kb
Mmp12 -10kb
Cd72 -11kb
Fcgr1 -7.5kb
Napepld -10kb
bGlob prom
g
ChIP SPT16
fold enrichment
WT
Trim33-/-
Napepld -10kb
Fcgr1 -7.5kb
bGlob prom
ChIP SPT16
Trim33-/-
Trim33-/- +WT TRIM33
h
3x sites PU.1 + +
FLAG-TRIM33 + +
PU.1 - +
FLAG
PU.1
β-ACTIN
ChIP FLAG-TRIM33
fold enrichment over IgG
FLAG-TRIM33+PU.1
FLAG-TRIM33
3x sites PU.1
i
Scale
chr9:
50 kb
mm9
96,300,000
96,350,000
Atp1b3
Rnf7
117 -
PU.1
0 _
301 -
TRIM33
0 _

FigureS2
