# Supplementary figures and images for: Interplay between FACT subunit SPT16 and TRIM33 can remodel chromatin at macrophage distal regulatory elements
Source: Epigenetics Chromatin. 2019 Jul 22;12:46. doi: 10.1186/s13072-019-0288-3 (PMC6647326; doi:10.1186/s13072-019-0288-3)

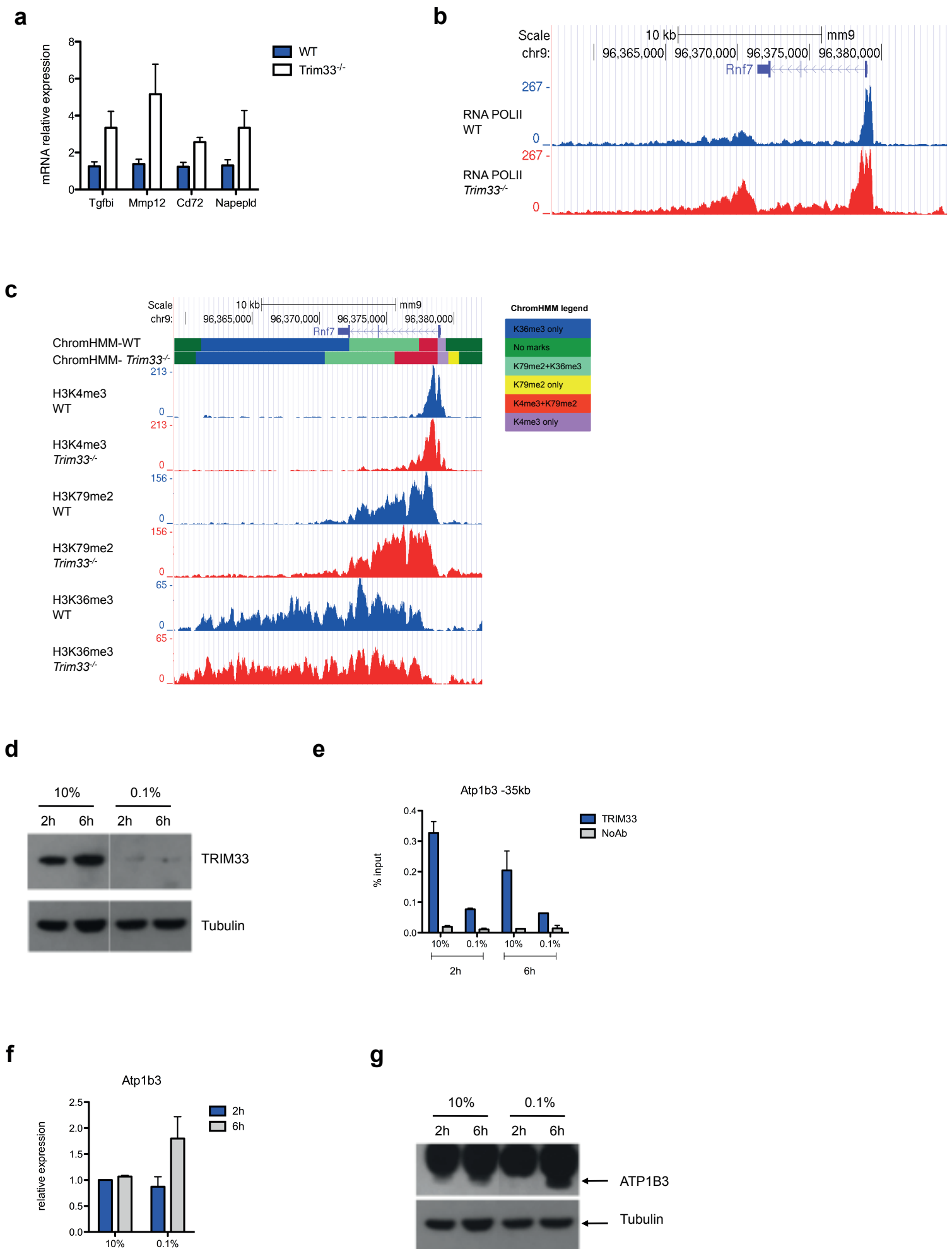

**FigureS3**

Supplement: Supplementary file 3 — Additional file 3: Figure S3. Related to Fig. 3. a mRNA levels of Tgfbi, Mmp12, Cd72 and Napepld in WT and Trim33−/− BMDM. Data are presented relative to expression of WT BMDM. Mean ± SEM, n = 4. b UCSC genome browser image of RNA Pol II at the Rnf7 gene in WT and Trim33−/− BMDM. c UCSC genome browser image of indicated chromatin modifications along with ChromHMM analysis at the Rnf7 gene in WT and Trim33−/− BMDM. d Immunoblotting showing TRIM33 expression in RAW cells grown in culture medium containing 10% or 0.1% FCS for 2 h and 6 h. e TRIM33 occupancy at the − 35 kb site in RAW cells grown in culture medium containing 10% or 0.1% FCS for 2 h and 6 h. Mean ± SEM, n = 2. f Atp1b3 mRNA expression levels in RAW cells grown in culture medium containing 10% or 0.1% FCS for 2 h and 6 h. Mean ± SEM, n = 2. g Immunoblotting showing ATP1B3 expression in RAW cells grown in culture medium containing 10% or 0.1% FCS for 2 h and 6 h [file 13072_2019_288_MOESM3_ESM.pdf]

**a**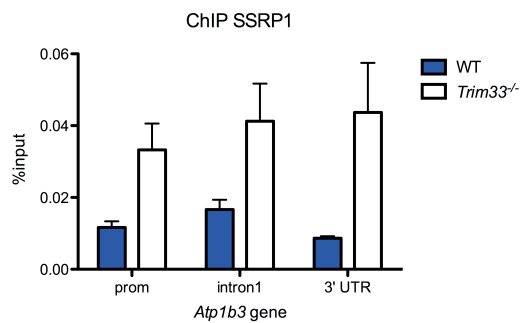**b**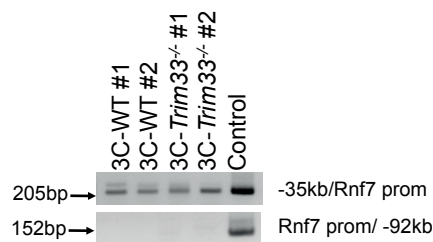**c**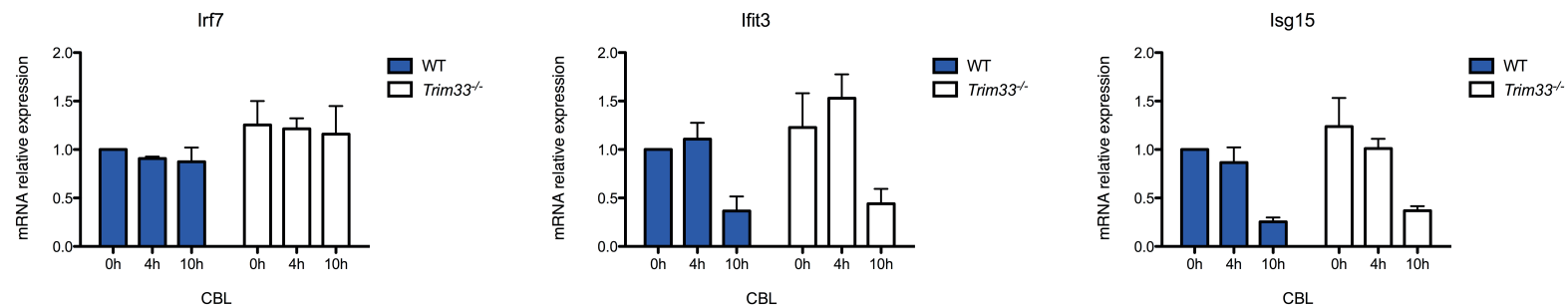**d**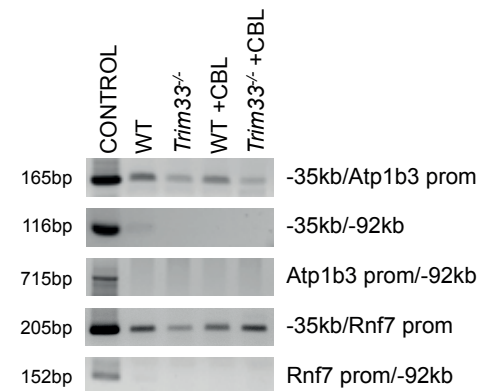**e**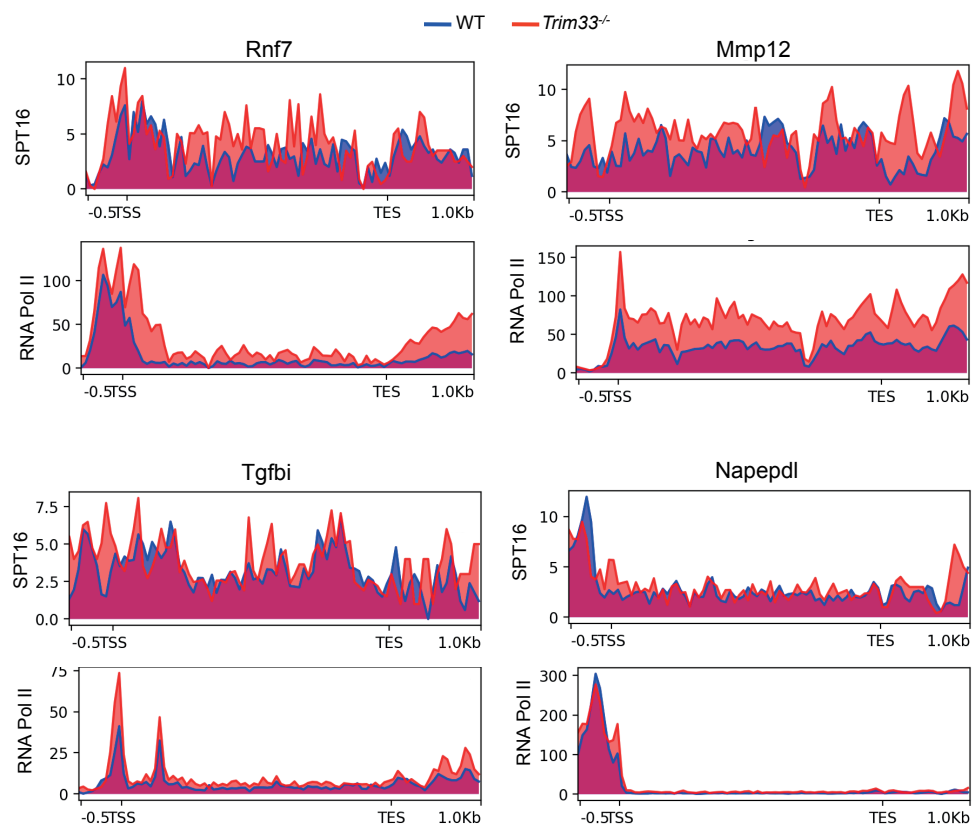**f**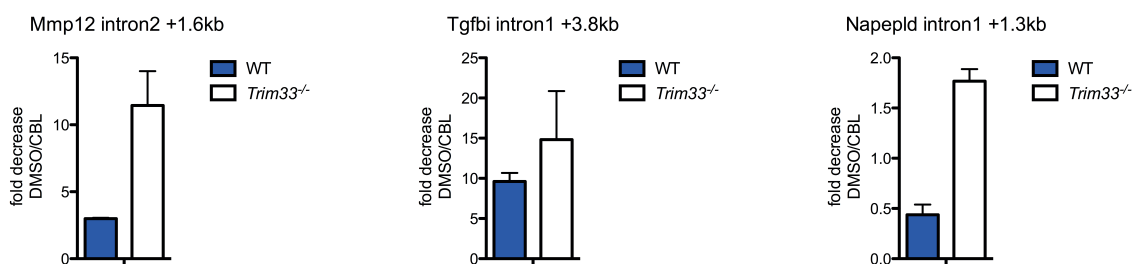

Supplement: Supplementary file 4 — Additional file 4: Figure S4. Related to Fig. 3. a SSRP1 ChIP-qPCR at the indicated positions in the Atp1b3 gene in WT and Trim33−/− BMDM. b 3C analyses of interaction between the − 35 kb region and the Rnf7 promoter. See Fig. 3g for the schematic of primers positions. c Kinetics of Irf7, Ifit3 and Isg15 mRNA relative expression in WT and Trim33−/− BMDM treated with CBL. Data are presented relative to expression of untreated WT BMDM. Mean ± SEM, n = 3. d 3C analyses at the Atp1b3/Rnf7 locus in WT and Trim33−/− BMDM treated for 4 h with CBL. See Fig. 3g for the schematic of primers positions. e SPT16 and RNA Pol II metaprofile analyses at indicated gene bodies in WT and Trim33−/− BMDM. f Fold decrease in WT and Trim33−/− BMDM, expressed as the ratio between DMSO and CBL-treated BMDM, of pre-mRNA levels of genes regulated by an intergenic SPT16/TRIM33 complex. Mean ± SEM, n = 3 [file 13072_2019_288_MOESM4_ESM.pdf]

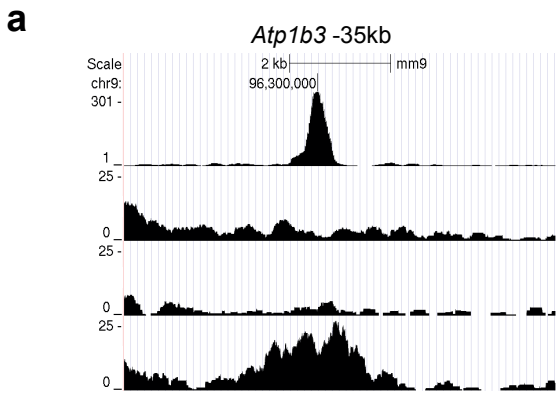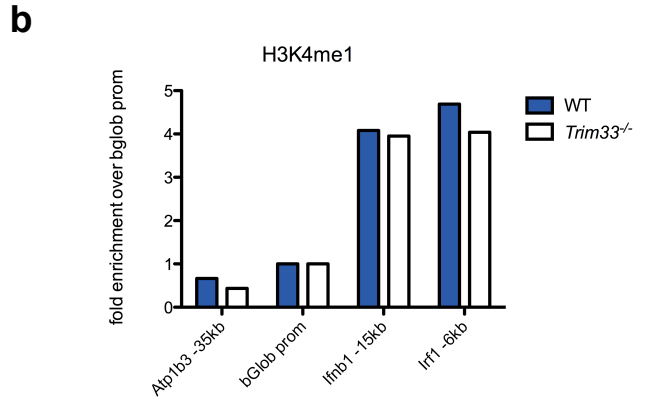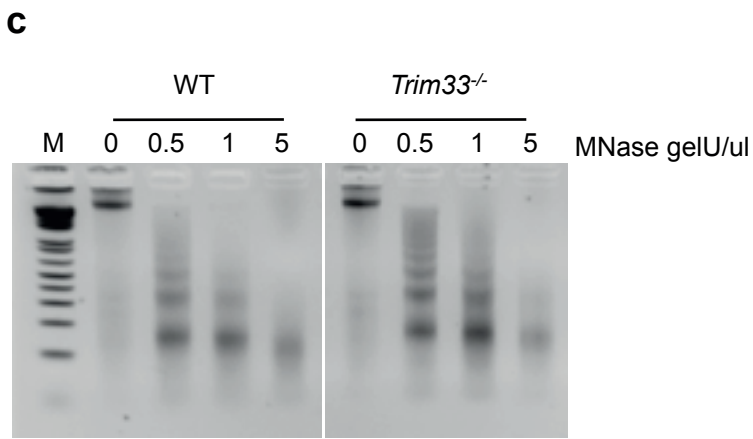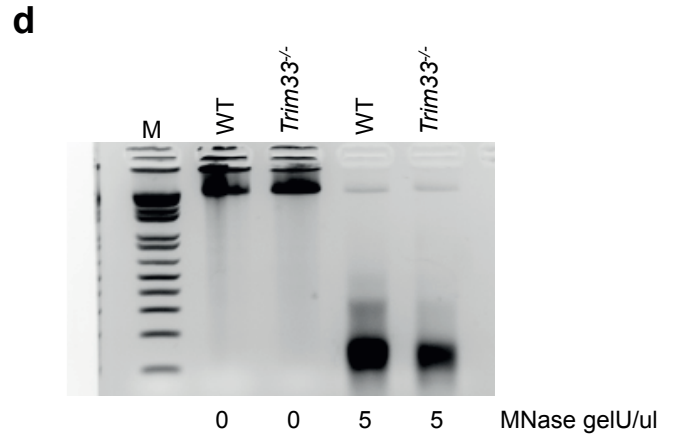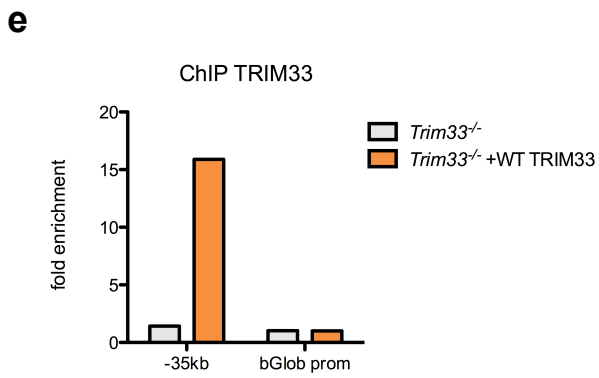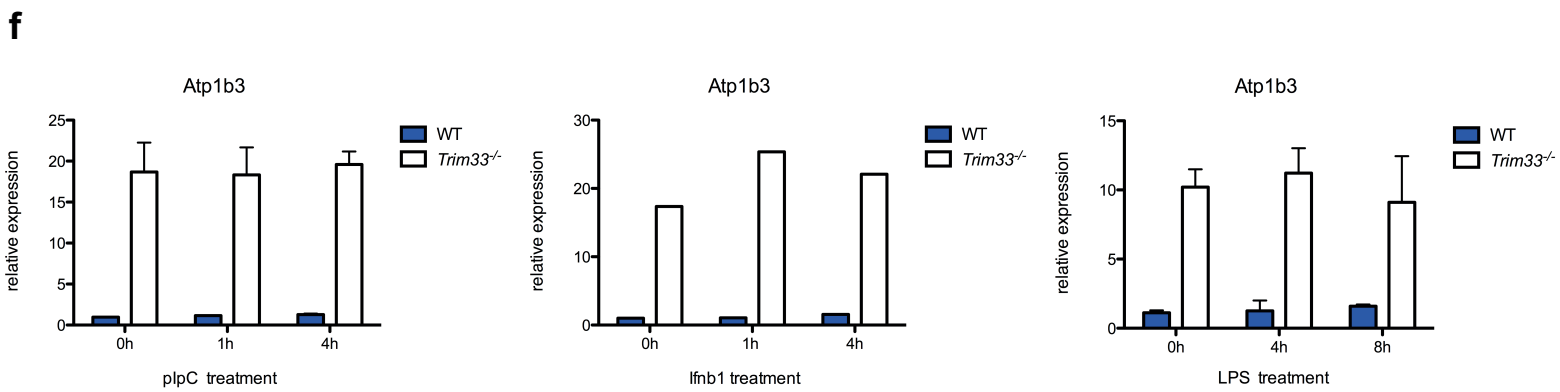

FigureS5

Supplement: Supplementary file 5 — Additional file 5: Figure S5. Related to Fig. 4. a H3K4me1, H3K4me2 and H3K27ac histone modification marks at the − 35 Kb region in BMDM. Also shown the TRIM33 peak. b H3K4me1 ChIP-qPCR at the − 35 kb region in WT and Trim33−/− BMDM. Regions near to Ifnb1 and Irf1 genes are used as positive controls and a region in the beta globin promoter is used as a negative control. c Representative image of MNase digestion profile. Nuclei from WT and Trim33−/− BMDM were digested with increasing amounts of MNase. d Representative image of MNase digestion profiles of chromatin from WT and Trim33−/− BMDM before mono-nucleosome purification. e TRIM33 ChIP-qPCR at the − 35 kb region in Trim33−/− and Trim33−/− + WT TRIM33 IM. f Kinetics of Atp1b3 mRNA levels in WT and Trim33−/− BMDM treated for the indicated times with pIpC (left), Ifnb1 (middle) and LPS (right). Data are presented relative to expression of untreated WT BMDM [file 13072_2019_288_MOESM5_ESM.pdf]
